# Understanding Complete Pathologic Response in Oesophageal Cancer: Implications for Management and Survival

**DOI:** 10.1155/2015/518281

**Published:** 2015-07-13

**Authors:** K. E. O'Sullivan, E. T. Hurley, J. P. Hurley

**Affiliations:** The Mater Private Hospital, Eccles Street, Dublin, Ireland

## Abstract

Despite significant improvement over recent decades, oesophageal cancer survival rates remain poor. Neoadjuvant chemoradiotherapy followed by oesophageal resection is mainstay of therapy for resectable oesophageal tumours. Operative morbidity and mortality associated with oesophagectomy remain high and complications arise in up to 60% of patients. Management strategies have moved towards definitive chemoradiotherapy for a number of tumour sites (head and neck, cervical, and rectal) particularly for squamous pathology. We undertook to perform a review of the current status of morbidity and mortality associated with oesophagectomy, grading systems determining pathologic response, and data from clinical trials managing patients with definitive chemoradiotherapy to inform a discussion on the topic.

## 1. Introduction

Oesophageal cancer is the 8th commonest malignancy worldwide affecting more than 450,000 people [1, 2]. Overall 5-year survival is estimated to be between 15 and 25% [1, 3]. While this appears low, significant improvements in treatment have been made in recent decades. Prognosis in the 1960s was considered so dismal that survival beyond two years was considered long-term and the rates were only 9% [4].

The two main histological subtypes are squamous cell carcinoma (SCC) and oesophageal adenocarcinoma (OAC), with rare variants such as spindle cell carcinoma, verrucous carcinoma, pseudosarcoma, mucoepidermoid carcinoma, adenosquamous carcinoma, primary oat cell carcinoma, choriocarcinoma, carcinoid tumour, leiomyosarcoma, malignant melanoma, rhabdomyosarcoma, and lymphoma making up 1-2% [5]. Whilst SCC is the most dominant type of esophageal cancer, the predominant histological subtype is highly variable depending on geographical location. Trends over time suggest that the overall incidence of SCC is stable or declining in westernized regions while, in contrast, OAC has overtaken SCC in most western countries with the incidence having increased >650% in the USA over the past 35 years [6].

SCC begins in squamous cells and is mainly found in the upper two thirds of the esophagus. Adenocarcinomas are derived from gland cells, which make up the mucus lining in the epithelium, typically in the lower third of the oesophagus. There are three grades of adenocarcinoma based on Siewert Classification [7]. Type 1 is adenocarcinoma of distal esophagus and type 2 is a true carcinoma of the cardia, with type 3 being subcardial gastric cancer infiltrating the distal oesophagus. Barrett's oesophagus (BO) is classified by specialized intestinal metaplasia of the lower oesophagus known to arise as a result of chronic gastroesophageal reflux disease (GORD) and ultimately the development of oesophagitis [8]. The condition is associated with an increased risk of cancer development and patients with oesophagitis have a relative risk of 4.5 for the development of cancer; this increases to 29.8 in those who have progressed to BO [9]. Many additional risk factors for oesophageal cancer exist including age, gender, race, obesity, reflux symptoms, smoking, and diet; however dysplasia remains the sole reliable indicator of adenocarcinoma development [10].

Oesophagectomy alone or with neoadjuvant chemoradiotherapy is the mainstay of therapy for nonmetastatic oesophageal cancer [11]. A randomized trial comparing chemotherapy followed by surgery with surgery alone in squamous cell carcinoma of the oesophagus found that medial overall survival time was 16 months in the chemosurgery group compared with 12 months in the surgery alone cohort [12]. Results of the MAGIC trial whereby patients were randomised to receive surgery alone or perioperative and postoperative chemotherapy with Epirubicin, Cisplatin, and Fluorouracil (ECF) demonstrated 36% 5-year survival for those in the chemotherapy group and 23% for those in the surgery only group [13]. The CROSS trial demonstrated a 49.4-month median overall survival in the chemoradiotherapy-surgery group compared to 24 months in the surgery alone cohort [14]. Approximately 25–30% of patients experience complete pathologic response following neoadjuvant chemoradiotherapy [14–16].

Even though the trimodal approach has led to prolonged survival outcomes, the risk of operative complications must be borne in mind [17]. A number of authors have therefore asked should every patient undergo oesophagectomy and how might the patients best suited to chemoradiotherapy alone be selected? We undertook to perform a review of the current grading systems pertaining to pathological response, the current levels of outcome data reporting pathologic response rates, and updated operative complication rates in order to inform a discussion on the topic.

## 2. Methodology

Relevant medical literature was identified from searches of PubMed and references cited in appropriate articles identified. Search terms included oesophageal adenocarcinoma, squamous cell carcinoma, complete pathological response, tumour regression grade, clinical trial oesophageal cancer, multimodal therapy oesophageal cancer, oesophagectomy morbidity, oesophagectomy mortality, and definitive chemoradiotherapy oesophageal cancer. Selection of articles was based on peer review, journal, and relevance. Where possible, articles from high-impact peer review journals were cited.

## 3. Current Tumour Regression Grading Systems

Attainment of complete pathological response is accepted as a surrogate marker of survival advantage; however no measure of histomorphological regression is included in the current staging nomenclature [18]. One reason is a lack of standardisation of tumour regression grading systems; a total of nine currently exist, 5 exclusively focusing on response at the primary site and 3 incorporating both nodal and primary site response (Table 1) [18–26]. The most frequently utilised regression grading system to date is that of Mandard et al. [22]. On the basis of gross examination, histology and oesophageal wall involvement primary tumour regression was classified into five histologic tumour regression grades with grade one demonstrating complete regression and an absence of histologically identifiable cancer and fibrosis extending through the different layers of the oesophageal wall and grade 5 with an absence of regressive changes [22]. The group estimated survival curves based on typical pathological features were associated with survival in addition to tumour regression grade and determined that after multivariate analysis regression grades 1–3 and 4-5 remained significant predictors of disease free survival, highlighting the importance of tumour regression in the survival of patients with oesophageal cancer undergoing preoperative chemoradiotherapy, suggesting that this should be taken into account when evaluating therapeutic results [22].

More recently, Donohoe et al. applied a comparative analysis of the existing tumour regression grading systems to a new 3-point tumour regression grade score using data from 393 consecutive patients undergoing multimodal therapy, demonstrating that only their novel score and not preexisting scores predicted survival [18]. The same authors argue that the difficulty with preexisting scoring systems is interpretation if the intermediate scores, for example, Mandard grades 2–4, whereby scoring presents difficulty with respect to interpretation of these intermediate grades with respect to interobserver differences [18]. Authors concluded that no existing published measure of tumour regression independently predicted outcome, compared with the novel 3-point score they devised. Despite the disparity between grading scores and the undoubted benefit obtainable from a consensus scoring system, any such score will not allow discrimination between those patients likely to derive benefit from nonoperative management.

Since publication of that paper, Lin et al. examined a pathologic complete response (pCR) nomogram score to predict the survival outcomes of patients receiving definitive chemoradiotherapy for oesophageal cancer [27]. Patients undergoing analysis (*n* = 333) had received chemoradiation without surgery for oesophageal cancer and a multivariate cox regression analysis was employed to test potential associations between clinical outcomes and patient or treatment factors [27]. A nomogram based on gender, endoscopic ultrasound findings, standard uptake value (SUV) after therapy, oesophagogastric duodenoscopy (OGD) post-neoadjuvant therapy, and histological tumour grade was devised. Interestingly, patients with a nomogram score falling below the median (<125) had significantly worse outcomes compared with those with scores above the median (>125). Furthermore, multivariate cox regression analysis indicated that nomogram score independently predicted each survival outcome, along with other patient and disease factors [27]. The group argues that although further validation of the nomogram score is required, it may prove useful for stratification of patients at highest risk of relapse and therefore requiring oesophageal resection.

Whilst a consensus tumour regression grading system is undoubtedly advantageous, it is not possible to apply that to the avoidance of surgery in those patients in whom a complete pathological response is obtained due to the fact that all of the existing grading systems utilise specimens resected at the time of surgery. A number of methods of predicting pCR have been explored, however. Skinner et al. have presented a validated miRNA signature to predict pCR to neoadjuvant chemoradiotherapy in oesophageal adenocarcinoma [28]. Three patient cohorts were examined: discovery (*n* = 10), model (*n* = 43), and validation (*n* = 65). In the discovery cohort 754 miRNA targets were examined in pretreatment tumour biopsies and of these 44 of the most significantly altered selected. The 4 miRNAs significantly predicting pCR in both the discovery and model group were further assessed in the validation cohort and these 4 miRNAs were used to generate an expression profile (MEP) score [28]. When combined with clinical variables, the MEP score provided a validated means of predicting pCR to neoadjuvant chemoradiotherapy [28].

A recent study by Wen et al. has examined whether mRNA markers are useful for the prediction of CRT in oesophageal squamous cell carcinoma [29]. Gene expression analysis was carried out on pretreatment biopsies from 28 patients who received neoadjuvant CRT and surgery. Authors derived a prediction model based on the qPCR values of three genes which was found to have a predictive accuracy of 86% after leave-out-one cross-validation. This model was then validated in another cohort of 32 patients with a predictive accuracy of 81%. Whilst larger validation studies are required, this may facilitate individualised therapy for oesophageal squamous cell carcinoma patients and allow discrimination between those likely to experience complete response compared to those who would benefit from oesophageal resection.

Biomarkers in peripheral blood are of interest as the predictors of response because blood collection is minimally invasive. Hsu et al. have recently reported identification of a serum biomarker FAM84B whereby reduced expression following neoadjuvant chemoradiotherapy was found to be significantly predictive of pCR [30]. The sample size for this study is small and the results require further validation in an independent cohort; nonetheless it represents a promising advance in the identification of more reliable predictors of complete pathological response in oesophageal squamous cell carcinoma capable of delineating those patients best suited to nonoperative management.

ALDH-1 has been recently identified as a universal marker of cancer stem cells and progenitor cells thought to result in tumour regeneration as a result of the somewhat dormant presence of a small population of dysregulated stem cells following chemoradiotherapy with the capability to revive and generate tumours capable of recapitulating the heterogeneity of the parent tumour [31, 32]. This concept was studied by Ajani et al. with regard to tumour regression who hypothesized that the presence of ALDH-1 could be associated with pathological response [33]. A cohort of 167 patients, 96% of whom had adenocarcinoma, were divided into those who had a pCR and those with extremely resistant cancer. For patients at extremes of this spectrum there was a significant association between pCR and low ALDH-1 levels analysed by immunohistochemistry with the inverse association demonstrated for patients with high ALDH-1 levels [33]. While this observation is undoubtedly useful, it falls short of being capable of predicting pCR in the entire cohort of patients with ambiguity regarding those with intermediate ALDH-1 expression levels. Furthermore, due to the small number of patients with squamous cell pathology, it is of uncertain benefit in this group.

Smit et al. have examined the same hypothesis in a murine model* in vivo*, examining xenograft tumours generated in NOD/SCID mice from a squamous cell carcinoma (OE21) and adenocarcinoma (OE33) cell line. CD44+/CD21− subpopulations were found to exert higher proliferation rates and more radioresistance* in vitro* compared with CD44+/CD24+ cells. Furthermore CD44+/CD24− cells formed xenograft tumours faster. This was partially validated in human tissue with the phenotype successfully identified in 50% of poor responders and none of the complete responders to neoadjuvant chemoradiotherapy.

As such, a predictive marker of complete pathological response in this setting remains elusive and will be a requirement for delineation of those patients for whom surgery represents additional unnecessary risk without additional survival benefit.

Certain pathological exceptions are likely to require individual attention. Signet ring morphology has consistently been associated with an unfavourable prognosis and response to neoadjuvant therapy is rare [34]. Patel et al. studied a group of 85 patients with signet ring cell adenocarcinoma compared with a 638-patient reference group and discovered a lower rate of complete pathological response (9 versus 26%, *P* < 0.001) and more frequent positive margins (24 versus 10%, *P* < 0.001) in the signet ring group [35]. Furthermore, survival duration was significantly decreased in the signet ring cohort and patients with signet ring histology whose resected specimens demonstrated significant downstaging after neoadjuvant therapy did not differ from the survival duration of those patients who did not exhibit downstaging unlike the reference group whose survival was improved [35].

## 4. Current Rates of Operative Morbidity and Mortality

Oesophageal cancer resection is associated with extremely high perioperative morbidity and mortality [36]. Neoadjuvant chemoradiation not only is now the gold standard therapy but has not been shown to increase postoperative morbidity or mortality [37, 38]. Furthermore, there appears to be no difference in the early or long-term survival between transthoracic and transhiatal oesophagectomy [39]. Factors known to increase operative risk include locally advanced disease, diabetes, dyspnoea, peripheral vascular disease and previous cerebrovascular accident, international normalised ratio, and wound contamination at the time of surgery [40]. While recent advances have been made with the introduction of standardised anaesthesia and surgical clinical pathways, lowering the incidence of complications remains a significant challenge [36, 41]. Adverse events are decreasing in relation to the treatment of many medical conditions; however their incidence with respect to surgery continues to increase [42].

Recognising a lack of standardisation of reporting strategies and definitions, a system for the definition and recording of complications and quality measures after oesophageal resection has been recently published, dividing complications into pulmonary, cardiac, gastrointestinal, urologic, thromboembolic, neurologic, infectious, and wound related categories; this has not yet been applied to the reporting of incidence; therefore data preceeding its publication is unreliable [41]. The introduction of minimally invasive operative approaches has again changed the operative paradigm and while they have been shown to lower morbidity and mortality, minor complications are still reported in 50–60% of patients [43–45]. Minimally invasive oesophagectomy is associated with a lower incidence of pulmonary complications and shorter hospital stay compared with the open approach [46]. One recently published series of robotic oesophagectomy reported rates of recurrent laryngeal nerve palsy in 26.3%, anastomotic leakage in 14.9%, and pulmonary complications in 9.6% [47]. Ninety-day mortality was 2.5% [47].

Anastomotic leak is a severe and life-limiting consequence of oesophagectomy occurring in 3–30% of patients and carrying a mortality rate of approximately 18.2% [14, 43, 48–50]. Reported rates are higher in those undergoing cervical anastomosis compared with thoracic such that routine radiological assessment of anastomotic integrity has been suggested for that patient cohort [51]. Presentation of intrathoracic oesophageal leaks ranges from patients who are asymptomatic to those with circulatory collapse and multiorgan failure [52]. Management is determined based on the size of the leak, extent of the abscess, and status of the patient with options including the “three-tube method” (thoracic closed drainage tube, gastrointestinal decompression tube, and enteral nutrition), self-expanding metallic stents, endoscopic vacuum assisted closure, and operative management [52, 53].

Recurrent laryngeal nerve palsy is reported to occur in 36% of cases, occasionally due to intentional excision as a result of metastatic node involvement, but does not seem to alter patient survival [54, 55]. Pulmonary complications are a frequent cause of morbidity in patients undergoing oesophagectomy and are reported to occur in approximately 35% of cases with an associated mortality of 6% [56]. Recent audits suggest hospital mortality rates of 7–9% [44]. Some centres report perioperative mortality rates as low as 1.1%, however [45]. The national 30-day readmission rate in the US following oesophagectomy is 12.6% with risk factors associated with readmission including a history of pulmonary disease, postoperative wound infections, and length of hospital stay prior to discharge [17].

## 5. Definitive Chemoradiotherapy for Oesophageal Cancer

Pathologic complete response (pCR) has been shown to predict decreased local and distant recurrence and improved survival in oesophageal cancer [20]. Current guidelines stipulate the necessity for operative resection in all patients who are fit to undergo surgery irrespective of the extent of tumour regression, leaving definitive chemoradiotherapy dCRT as an alternative for those patients unsuitable for surgery due to comorbid disease [57]. In recent years the concept of definitive chemoradiotherapy has emerged as a primary treatment option for a number of squamous cell carcinomas in sites such as cervix and head and neck where salvage surgery is reserved for those with disease relapse [58, 59]. Furthermore, there have been several recent reports on a nonoperative approach for lower rectal cancers [60].

A number of studies to date have reported long term survival outcome following definitive chemoradiotherapy for patients with resectable squamous cell carcinoma of the oesophagus. A definitive answer as to whether this approach offers similar cure rates with decreased morbidity and mortality has not yet been obtained. Teoh et al. randomized 81 patients with resectable mid- or lower oesophageal squamous cell carcinoma to receive oesophagectomy or dCRT [61]. Although the overall 5-year survival did not reach statistical significance it did favour the dCRT group (surgery 29.4%, dCRT 50%, *P* = 0.147) and the 5-year disease-free survival also showed a trend towards favouring dCRT (*P* = 0.068) [61]. The RTOG 85-01 trial set the definitive standard for dCRT in 1992 [62]. This phase III prospective, randomized trial evaluated the efficacy of four courses of fluorouracil and cisplatin plus 5,000 cGy of radiation therapy compared with 6,400 cGy of radiation therapy alone in patients with adenocarcinoma (*n* = 15) or squamous cell carcinoma (*n* = 106) of the oesophagus and was stopped after accumulation of results from 121 patients when a significant survival advantage for those receiving chemoradiotherapy was identified [62]. Median survival for those receiving radiation was 8.9 months compared with 12.5 months. Long-term follow-up showed that 5-year survival was 26% for chemoradiotherapy and 0% for radiotherapy alone. In those receiving chemoradiotherapy, persistent disease and locoregional relapse were the main causes of treatment failure [62]. A follow-up trial (INT 0123) showed that a higher dose of radiotherapy (64.8 Gy) was not superior in terms of survival or disease control compared with a lower dose (50.4 Gy) but was more toxic [63].

In Europe the use of CRT in the neoadjuvant setting became more widespread after the publication of several meta-analyses demonstrating a benefit [64–66]. Stahl et al. performed a trial for locally advanced SCC of the upper/mid oesophagus, allocating patients to induction chemotherapy followed by CRT (40 Gy) with surgery or induction chemotherapy followed by CRT (65 Gy) without surgery [67]. Overall survival did not improve with the addition of surgery; however patients in the surgery arm did experience improved local control and a reduction in death secondary to the cancer [67]. However, this trial was designed to assess equivalence and not survival advantage [67].

Cisplatin and fluorouracil represent the mainstay of chemotherapy although other choices have been examined including FOLFOX versus fluorouracil and cisplatin, cisplatin/irinotecan versus carboplatin/paclitaxel, paclitaxel, carboplatin, carboplatin, and paclitaxil (CROSS protocol) [14, 68–70]. Survival rates were similar with FOLFOX compared with cisplatin and fluorouracil; however the side effect profile was slightly different with increased neuropathy and less renal dysfunction with FOLFOX [68].

Two trials comparing the use of preoperative CRT followed by surgery versus dCRT have been conducted with almost exclusively squamous cell histology. The German trial, exclusively SCC, used induction chemotherapy (fluorouracil, leucovorin, etoposide, and cisplatin for 3 cycles) followed by chemoradiotherapy (40 Gy with cisplatin and etoposide) followed by either surgery or further radiotherapy [67]. In the French trial 89% of patients had squamous pathology and received either split-course radiotherapy (30 Gy) or standard radiotherapy (46 Gy) with two cycles of cisplatin and fluorouracil [71]. Only patients experiencing a response after induction chemotherapy were randomized; half to further chemoradiation and half to surgery. Both trials were designed to assess equivalence. Overall survival at two years was similar in both arms of both trials. Local control rate at two years was better for surgery in both trials (66.7% for preoperative CRT versus 57% for dCRT in the French trial and 64.3% for preoperative CRT and 40.7% for dCRT in the German trial) [67, 71].

SCOPE 1 was a multicentre UK phase II-III trial comparing dCRT (50 Gy in 25 fractions with four cycles of cisplatin/capecitabine) with dCRT and the addition of cetuximab, an EGFR antagonist [72]. Patients included a total of 65 with adenocarcinoma, 188 with squamous, and 5 with undifferentiated pathology. Recruitment was stopped without continuation to phase III because the trial met criteria for futility. Fewer patients were treatment failure free at 24 weeks in the CRT plus cetuximab arm than in the CRT alone group and they also had a shorter median survival (22.1 months versus 25.4 months *P* = 0.035) [72]. Although the findings with regard to EFGR inhibitor therapy were negative, the overall survival for dCRT using modern strategies for patient selection and therapy delivery was better than pervious published trials of neoadjuvant CRT and surgery [72].

The issue, however, remains unresolved and a study has been performed to identify the feasibility of performing a randomized controlled trial comparing neoadjuvant CRT and dCRT in the UK [73]. Authors concluded that the numbers of incident eligible patients would be too low to enable such a study [73]. In patients with squamous cell histologies, CRT alone with careful follow-up and salvage surgery may be a reasonable approach for those who attain a pCR; however the paucity of data outrules any reasonable consideration of this approach in adenocarcinoma [74]. NCCN Guidelines recommend the use of dCRT for T4b tumours in medically fit and unfit patients and for resectable tumours in medically unfit patients. In medically fit patients with resectable tumours, dCRT is only recommended for cervical SCC oesophageal cancers and for all patients with adenocarcinoma it is only recommended for those who decline or cannot withstand surgery [75].

Belgian guidelines recommend that the use of dCRT be restricted to clinical trials for those patients with resectable disease unless diagnosed with cervical SCC oesophageal cancers [74]. Considering the body of evidence acquired to date and the known morbidity and mortality associated with oesophageal resection, it becomes apparent that clinical trials addressing this issue should be a priority and further information is required to select those patients who might be more appropriately managed with dCRT.

## 6. Future Considerations

The viability of dCRT with surgery reserved for those patients exhibiting a poor response is entirely dependent on an accurate method of determining true pCR, which is currently lacking. There are some promising mRNA studies; however as mentioned they require validation. Studies examining the accuracy of PET-CT in assessing response in oesophageal adenocarcinoma have revealed no significant association between maximum standard uptake value (SUV) and tumour regression grade [76]. Of a one hundred patient cohort examined, 80% had histological evidence of residual tumour in the resected specimen and a complete metabolic response was not associated with a survival benefit [76]. A cohort of 57 patients were examined by Klayton et al., who identified that while PET imaging was useful in terms of predicting the likelihood of residual tumour it was not sensitive enough to outrule the presence of residual disease [77]. These results are mirrored by those of Myslivecek et al. who found no statistically significant correlation between the (18)F-FDG metabolic response after neoadjuvant CRT and the histopathologic response in a cohort of 73 patients [78]. In a small series of patients undergoing neoadjuvant CRT for squamous cell carcinoma of the oesophagus, however, Park et al. did identify a relationship between change in SUV and likelihood of a complete response indicating that it may be more useful in this setting [79]. PET-CT is insufficient alone to determine a complete clinical response. Cheedella et al. examined 284 patients with oesophageal cancer and found that while 77% achieved a postneoadjuvant therapy biopsy negative, PET negative status, and therefore a complete clinical response, only 31% of patients in fact achieved a pCR following resection [80]. A recently published study by Kukar et al. examining the role of PET-CT characteristics in oesophageal adenocarcinoma patients has demonstrated that a change in SUV value less than 45% is associated with patients with residual disease but not complete pathological response [81]. No altered recommendation with respect to surgical resection is possible based on PET SUV in the primary tumour [81].

The possibility that SUV is a suboptimal parameter for classification has been explored. A group of 45 patients examined by Metser et al. evaluated tumours based on PET response criteria in solid tumour, based on criteria including SUL (standardized uptake value normalised to lean body mass), SUL tumour/liver ratio, and % change in SUL. Results demonstrated a positive correlation between posttherapy SUL ratio, % change in SUL, and % change in SUL ratio with clinical response (*P* = 0.025, 0.035, and 0.03, resp.); however further validation of this is required [82].

## 7. Conclusion

Despite surgical advances and more minimally invasive approaches, morbidity and mortality associated with oesophagectomy remain high. Definitive CRT has been used for patients unfit to undergo operative resection for many years and response rates have improved with the development of contemporary therapeutic regimens. With movement to more targeted systemic treatments it is likely that rates of pCR will increase. Accurate assessment of tumour response and regression without the information attained at pathological assessment postresection remains a significant challenge. Current modalities, endoscopy and PET-CT, are not sufficiently sensitive to allow reliable determination of complete response. In the future, research to identify those patients who may not benefit from oesophagectomy is warranted, particularly the identification of circulating biomarkers which predict disease response.

## Figures and Tables

**Table 1:** Tumour response grading systems.

Author	Complete response	Incomplete response	No response
*Tumor response grading systems that incorporate nodal response and primary site *			
Meredith et al. [23]	Complete response: absence of histological evidence of neoplasia, gross tumor, or individual cells in the resected esophageal specimen by light microscopy but not immunohistochemical stains	Partial response: change in T or N stage from preoperative EUS or greater than 50% reduction in size of tumor compared pre- and postoperatively	No response: no change in tumor stage compared to preoperative EUS stage and postoperative pathology stage
Donahue et al. [21]	Complete response: microscopic absence of any viable tumor	Near-complete: microscopic focus of viable tumor cells in an otherwise necrotic specimen with no tumor remaining in resected lymph nodes	No response: macroscopic residual viable tumor at primary site and/or positive lymph nodes
Kim et al. [24]	No residual tumor	Residual tumor <1 cm in greatest dimension and limited to mucosa or submucosa with no nodal involvement or primary and microscopic neoplastic cells in a single regional node	No response: all other tumors

*Tumor response grading systems of response at primary site only *			
Schneider et al. [25]	0 vital residual tumor cells at primary site	<10% vital residual tumor cells at primary site 10%–50% vital residual tumor cells at primary site	>50% vital residual tumor cells at primary site
Chirieac et al. [20]	Complete response: no residual cancer cells	Partial response: 1%–50% residual tumor	No response: more than 50% of tumor remains
Mandard et al. [22]	Grade 1: complete regression	Grade 2: isolated cell nests Grade 3: more residual cancer cells but fibrosis still predominates Grade 4: residual cancer	Grade 5: absence of regressive changes
Barbour et al. [19]	N/A	Major response: <10% residual viable tumor cells	>10% residual viable tumor cells
Donington et al. [26]	No vital residual tumor cells at primary site		Any residual tumor cells at primary site
